# Effects of a Low Dose of Caffeine Alone or as Part of a Green Coffee Extract, in a Rat Dietary Model of Lean Non-Alcoholic Fatty Liver Disease without Inflammation

**DOI:** 10.3390/nu12113240

**Published:** 2020-10-23

**Authors:** Ana Magdalena Velázquez, Núria Roglans, Roger Bentanachs, Maria Gené, Aleix Sala-Vila, Iolanda Lázaro, Jose Rodríguez-Morató, Rosa María Sánchez, Juan Carlos Laguna, Marta Alegret

**Affiliations:** 1 Department of Pharmacology, Toxicology and Therapeutic Chemistry, School of Pharmacy and Food Science, University of Barcelona, Avda Joan XXIII 27-31, 08028 Barcelona, Spain; avelazquezpy@gmail.com (A.M.V.); roglans@ub.edu (N.R.); bentanachs96@gmail.com (R.B.); mariagene_15@hotmail.com (M.G.); rmsanchez@ub.edu (R.M.S.); 2 Institute of Biomedicine, University of Barcelona, 08028 Barcelona, Spain; 3 Spanish Biomedical Research Centre in Physiopathology of Obesity and Nutrition (CIBEROBN), Instituto de Salud Carlos III (ISCIII), 28029 Madrid, Spain; jose.rodriguez@upf.edu; 4 IMIM-Hospital del Mar Medical Research Institute, 08003 Barcelona, Spain; asala@barcelonabeta.org (A.S.-V.); iolan.lazaro@gmail.com (I.L.); 5 Barcelonaβeta Brain Research Center, Pasqual Maragall Foundation, 08005 Barcelona, Spain; 6 Department of Experimental and Health Sciences, Universitat Pompeu Fabra (CEXS-UPF), 08003 Barcelona, Spain

**Keywords:** caffeine, coffee, dietary supplements, hepatic steatosis, non-alcoholic fatty liver disease

## Abstract

Non-alcoholic fatty liver disease is a highly prevalent condition without specific pharmacological treatment, characterized in the initial stages by hepatic steatosis. It was suggested that lipid infiltration in the liver might be reduced by caffeine through anti-inflammatory, antioxidative, and fatty acid metabolism-related mechanisms. We investigated the effects of caffeine (CAF) and green coffee extract (GCE) on hepatic lipids in lean female rats with steatosis. For three months, female Sprague-Dawley rats were fed a standard diet or a cocoa butter-based high-fat diet plus 10% liquid fructose. In the last month, the high-fat diet was supplemented or not with CAF or a GCE, providing 5 mg/kg of CAF. Plasma lipid levels and the hepatic expression of molecules involved in lipid metabolism were determined. Lipidomic analysis was performed in liver samples. The diet caused hepatic steatosis without obesity, inflammation, endoplasmic reticulum stress, or hepatic insulin resistance. Neither CAF nor GCE alleviated hepatic steatosis, but GCE-treated rats showed lower hepatic triglyceride levels compared to the CAF group. The GCE effects could be related to reductions of hepatic (i) mTOR phosphorylation, leading to higher nuclear lipin-1 levels and limiting lipogenic gene expression; (ii) diacylglycerol levels; (iii) hexosylceramide/ceramide ratios; and (iv) very-low-density lipoprotein receptor expression. In conclusion, a low dose of CAF did not reduce hepatic steatosis in lean female rats, but the same dose provided as a green coffee extract led to lower liver triglyceride levels.

## 1. Introduction

Non-alcoholic fatty liver disease (NAFLD) is a spectrum of alterations ranging from simple hepatic steatosis to non-alcoholic steatohepatitis (NASH), cirrhosis, and hepatocellular carcinoma. Hepatic steatosis, defined as the accumulation of triglycerides (TGs) in lipid droplets in at least 5% of the hepatocytes, is the initial reversible phase of NAFLD, affecting around 33% of adults in the US [1]. Although NAFLD is usually associated with obesity, this condition might also be present in individuals with a body mass index in the normal range, which is referred to as lean or non-obese NAFLD [2]. Compared to obese NAFLD, lean individuals with NAFLD are more commonly female and exhibit a lower prevalence of insulin resistance [2,3].

Consumption of sweetened beverages with a high fructose content is one of the main dietary triggers of NAFLD [4]. Despite the implementation of public policies that aim to reduce their consumption, a recent study on diet population trends showed that 42% of energy intake in US adults still comes from low-quality carbohydrates, including fruit juices and added sugars in beverages [5]. Moreover, the consumption of saturated fats, another dietary factor associated with NAFLD, still remains above the recommended maximal intake of 10% of the energy intake [5].

Given the difficulty to avoid excessive consumption of simple sugars and fats in the population, one strategy to fight NAFLD is the inclusion in the usual diet of functional foods or dietary supplements that could be effective to prevent or reduce hepatic lipid accumulation. Several meta-analysis of randomized clinical trials showed that compounds such as resveratrol, silymarin, vitamin E or D, and curcumin, exert positive effects on NAFLD, which might be attributed to their antioxidant or anti-inflammatory properties [4]. However, not all evidences showed clinical efficacy, which could be related to the different doses, formulation issues, or duration of studies [6,7,8]. Coffee was reported to exert beneficial effects on liver-related disorders [9], including a reduced risk of NAFLD and of liver fibrosis in NAFLD patients, as revealed by a recent meta-analysis [10]. Effects of coffee on NAFLD development were mainly ascribed to its caffeine content. Several studies indicated that caffeine reduces intrahepatic fat accumulation in mice and rats, however, these studies did not specify the dose of caffeine based on animal weight or they used a dose close to the maximal one admitted in humans after interspecies conversion [11,12,13]. Moreover, coffee contains more than one hundred compounds besides caffeine, and it is especially rich in polyphenols such as chlorogenic acids [14], which might also be responsible for its beneficial effects.

In the present study, we investigated the effects of a moderate dose of caffeine (5 mg/kg/day, alone or as part of a green coffee extract) in a model of hepatic steatosis without obesity and without inflammation, induced in female rats by feeding a cocoa butter-rich, high-fat diet, together with liquid fructose. Female rats were used, as non-obese steatosis is more frequent in females than in males [3]. The aims of the study were to determine whether caffeine at this low dose reversed hepatic steatosis in this model, whether there were different effects when the same dose of caffeine was administered in the form of a coffee extract, and to explore the mechanisms involved.

## 2. Materials and Methods

### 2.1. Animals

Female Sprague Dawley rats were purchased from Envigo (Barcelona, Spain). Animals were maintained under conditions of constant humidity (40–60%) and temperature (20–24°C), with a light/dark cycle of 12 h (2 rats/cage). Studies were conducted in accordance with the principles and procedures outlined in the guidelines established by the Bioethics Committee of the University of Barcelona (Autonomous Government of Catalonia Act 5/21 July 1995). The Animal Experimentation Ethics Committee of the University of Barcelona approved all experimental procedures involving animals (approval no. 10106).

### 2.2. Dosage Regimen

Forty-eight female rats aged 8 weeks were randomly assigned into 4 groups (*n* = 12 in each), which received: (1) standard rodent chow (control group, CT); (2) high-fat diet, and 10% w/v fructose in the drinking water (high-fat-high-fructose group, HF-HFr); (3) high-fat diet containing caffeine (from Sigma–Aldrich, St. Louis, MO, USA, 0.18 g/kg of diet) and 10% w/v fructose in drinking water (caffeine group, CAF); or (4) high-fat diet containing a green coffee extract providing 0.18 g of caffeine/kg of diet, and 10% w/v fructose in the drinking water (green coffee extract group, GCE). Groups 1 and 2 received their respective diets for 3 months. Groups 3 and 4 received the HF-HFr diet for 2 months, with the caffeine or green coffee extract supplied to the rats incorporated in the high-fat diet pellets during the third month of the protocol. The green coffee extract (a generous gift from Applied Food Science Inc., Austin, TX, USA) was obtained by extraction with 70:30 ethanol:water mixture, and then the extract was filtered, evaporated, and spray dried. The compositions of the control diet (2018 Teklad Global 18% protein) and the high-fat diet (Teklad Custom Diet TD.180456) are detailed in Supplementary material Table S1. Diets containing caffeine and green coffee extract were prepared by Envigo (Madison, WI, USA), by mixing the compounds with the different ingredients of the high-fat diet and pelleting. Fructose solutions were changed every two days. Throughout the treatment, solid food and liquid consumption was controlled three times a week, and body weight was recorded once a week. Based on the amount of diet consumed and the body weight of each rat, the amount of caffeine ingested in both the CAF and GCE groups was 5.0 ± 0.8 mg/kg/day. The human equivalent dose based on body surface area (K_m_ value for humans = 37 and for rats weighing 250 g = 7) was 0.95 mg/kg/day [15].

### 2.3. Open Field Test

In the last week of the treatment, an open field test (OFT) was performed to study locomotor activity in the control and treated rats. Rats were placed in the middle of a black box (40 × 40 × 40 cm), under a low illumination of 12 lux. Rats underwent habituation sessions for two consecutive days. On the third day, the distance traveled by each rat was monitored during 60 min (SMART^®^ version 3.0 software, Panlab SL, Barcelona, Spain). The OFT apparatus was cleaned with 10% ethanol solution, before using it with another rat.

### 2.4. Oral Glucose Tolerance Test

An oral glucose tolerance test (OGTT) was performed in the last week of the treatment, one day after the OFT test. Rats were fasted for 6 h, and a sample of blood was collected from the tail vein (time 0). A glucose solution of 2 g/kg of body weight was then administered by oral gavage, and blood samples were collected from the tail vein at 15, 30, 60, and 120 min after glucose administration. Glucose levels were determined in all blood samples using a hand-held glucometer (Accutrend^®^ Plus System, Cobas, Roche Farma, Barcelona, Spain). Plasma was obtained from blood samples collected at 0, 15, and 120 min, and insulin levels were measured using a rat insulin enzyme-linked immunosorbent assay (ELISA) kit (Millipore, Billerica, MA, USA).

### 2.5. Sample Preparation

At the end of the treatment, rats were fasted for 2 h and blood samples were obtained from the tail vein to measure TG, cholesterol, and glucose levels, using an Accutrend^®^ Plus system glucometer (Cobas, Roche Farma, Barcelona, Spain). The rats were then immediately anesthetized with ketamine/xylazine (9 mg/40 μg per 100 g of body weight, respectively) and blood was collected into micro-tubes (Sarstedt AG ^&^ Co., Nümbrecht, Germany) through cardiac puncture and centrifuged at 10,000× *g* for 5 min, at room temperature. Rats were euthanized by exsanguination, and the liver and visceral white adipose tissue (vWAT) were collected and weighed. For the histological studies, samples of the liver of each animal were fixed in buffered formalin or were embedded in OCT, frozen quickly in liquid nitrogen, and stored at −80 °C. The remaining liver tissues were immediately frozen in liquid nitrogen and stored at −80 °C until needed for biomolecular assays.

### 2.6. Plasma Analysis

Plasma samples were assayed in duplicates. Insulin and adiponectin concentrations were determined using specific ELISA kits (Millipore, Billerica, MA, USA). Alanine aminotransferase (ALT) activity was determined using an ALT/GPT enzymatic assay kit (Spinreact, Girona, Spain). Insulin sensitivity index (ISI) was calculated as 2/[plasma insulin (nM) × blood glucose (µM) + 1].

### 2.7. Histological Studies

Liver samples were dehydrated and paraffin embedded using a Leica TP1020 automatic tissue processor and a Leica EG1150 H Paraffin Embedding Module (Leica Microsistemas, Barcelona, Spain). Samples were cut to 5 microns and stained with hematoxylin and eosin (H&E). Lipid accumulation was analyzed in OCT-embedded liver sections stained with Oil-Red O (ORO). Images were acquired with a Leica DMSL microscope equipped with a DP72 camera (Leica Microsistemas, Barcelona, Spain) and analyzed using Image J 1.49 software (National Institutes of Health, Bethesda, MD, USA). The area of positive ORO staining was calculated as the positively stained area per total area. All procedures were carried out in the Animal Histopathology Laboratory at the University of Barcelona.

### 2.8. Liver Assays

Liver TGs were extracted as described by Qu et al. [16] and determined using a TG colorimetric assay kit (Spinreact, Girona, Spain). Total hepatic fatty acid β-oxidation was determined in rat livers, as described by Lazarow [17], using 30 μg of postnuclear supernatant.

#### 2.8.1. Measurement of Fatty Acid Methyl Esters in Liver TGs

Fatty acid methyl esters (FAMEs) from liver TGs were determined by gas chromatography/electron ionization mass spectrometry as described in the Supplementary Methods and Table S3.

#### 2.8.2. Lipidomic Analysis in Rat Liver Homogenates

Levels of diacylglycerols [DAG], ceramides [Cer], and hexosylceramides [HexCer] in rat livers were determined by liquid chromatography-tandem mass spectrometry (LC–MS/MS) system, as described in Supplementary Methods and Table S4.

### 2.9. RNA Preparation and Analysis

Total RNA was isolated from the liver samples using Trizol^®^ (Invitrogen, Carlsbad, CA, USA), cDNA was synthesized by reverse transcription and specific mRNAs were assessed by real-time reverse transcription polymerase chain reaction (RT-PCR), as described previously [18]. TBP (TATA-box-binding protein) was used as an internal control. The primer sequences and PCR product lengths are listed in Supplementary Material (Table S2).

### 2.10. Preparation of Protein Extracts

Liver samples were homogenized with a Polytron PT 1200E in lysis buffer containing proteases, phosphatases, and deacetylase inhibitors, and incubated for 1.5 h at 4 °C. Samples were then centrifuged at 15,000× *g* for 15 min at 4 °C, and the supernatants were collected. To obtain hepatic nuclear extracts, samples were homogenized with a homogenization buffer, kept on ice for 10 min, and centrifuged at 1000× *g* for 10 min at 4 °C. Lysis buffer was added to the obtained pellet and samples were incubated for 1.5 h at 4 °C, before being centrifuged at 25,000× *g* for 30 min at 4 °C. The resulting supernatants were then collected. Protein concentrations were determined by the Bradford method [19].

### 2.11. Western Blot Analysis

Western blots were performed using three samples per group, each sample was pooled from two animals. A total of 20–30 μg of protein extracts were subjected to SDS-polyacrylamide gel electrophoresis. Proteins were then transferred onto Immobilon polyvinylidene difluoride transfer membranes (Millipore, Billerica, MA, USA), and blocked for 1 h at room temperature, with 5% non-fat milk solution in Tris-buffered saline (TBS) containing 0.1% Tween-20. Membranes were then incubated with specific primary antibodies. Detection was performed using the Immobilion Western HRP substrate Peroxide Solution^®^ (Millipore, Billerica, MA, USA). To confirm the uniformity of protein loading, blots were incubated with anti-β-actin or anti-β-tubulin antibody (Sigma–Aldrich, St. Louis, MO, USA) as a control for total protein extracts, and with anti-TBP antibody (AbCam, Cambridge, UK) for nuclear protein extracts.

### 2.12. Statistical Analysis

The results are expressed as mean ± standard deviation (SD). Significant differences between the groups were established by one-way ANOVA and Šidák’s post-hoc test for selected comparisons (GraphPad Software version 8, San Diego, CA, USA). When the SD of the groups was different according to Bartlett’s test, the data were transformed into their logarithms and ANOVA was rerun, or the corresponding non-parametric test was applied. The OGTT curves for glucose and insulin were analyzed by two-way ANOVA. The level of statistical significance was set at *p* ≤ 0.05.

## 3. Results

### 3.1. The HF-HFr Diet Does Not Induce Obesity or Gluconeogenic Gene Expression

As shown in Table 1, although the HF-HFr diet induced a 1.8-fold increase in total caloric intake, the final body weight and vWAT weight were not significantly modified. Only the liver weight/body weight ratio showed a significant increase in response to the HF-HFr diet. Locomotor activity (measured as the total distance travelled in the open field test) was not significantly affected by the diet or treatments.

**Figure 1:**
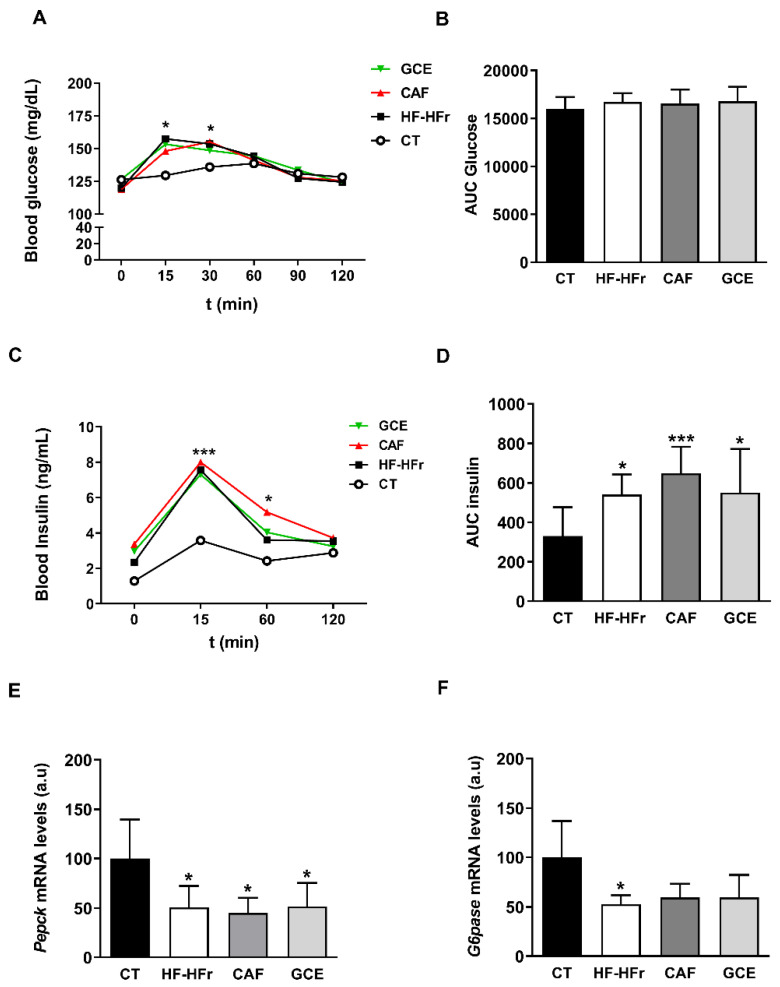
Blood glucose (**A**), area under the curve (AUC) for glucose (**B**), plasma insulin (**C**), and AUC for insulin (**D**) at different times after oral administration of a glucose solution (2 g/kg body weight). Results are the mean ± SD of values from 10–12 animals/group. Bar plots representing the mean ± SD mRNA levels corresponding to liver *Pepck* (**E**) and *G6Pase* (**F**) genes from CT (*n* = 5), HF-HFr (*n* = 6), CAF (*n* = 6) and GCE (*n* = 6) experimental groups. * *p* < 0.05; *** *p* < 0.001 vs. control.

Basal blood glucose and insulin levels were similar across the different groups (Table 1). After a glucose challenge in the OGTT, all groups on the HF-HFr diet exhibited higher glucose levels than the CT group at the shortest time points (Figure 1A). However, no differences were observed in the integrated glucose concentration, which was calculated as the area under the curve (AUC) (Figure 1B). Both the insulin levels (Figure 1C) and the corresponding AUC (Figure 1D) were significantly increased by the HF-HFr diet, with neither CAF nor GCE attenuating this increase. Accordingly, the ISI was significantly reduced in the HF-HFr group and none of the treatments reversed this decrease (Table 1). The mRNA levels of the insulin-responsive gluconeogenic genes phosphoenolpyruvate carboxykinase (*Pepck*) and glucose-6 phosphatase (*G6Pase*) decreased in the rats fed the HF-HFr diet (Figure 1E–F).

### 3.2. GCE Exerts Different Effects Compared to CAF on Hepatic TG Amount and Composition

**Figure 2:**
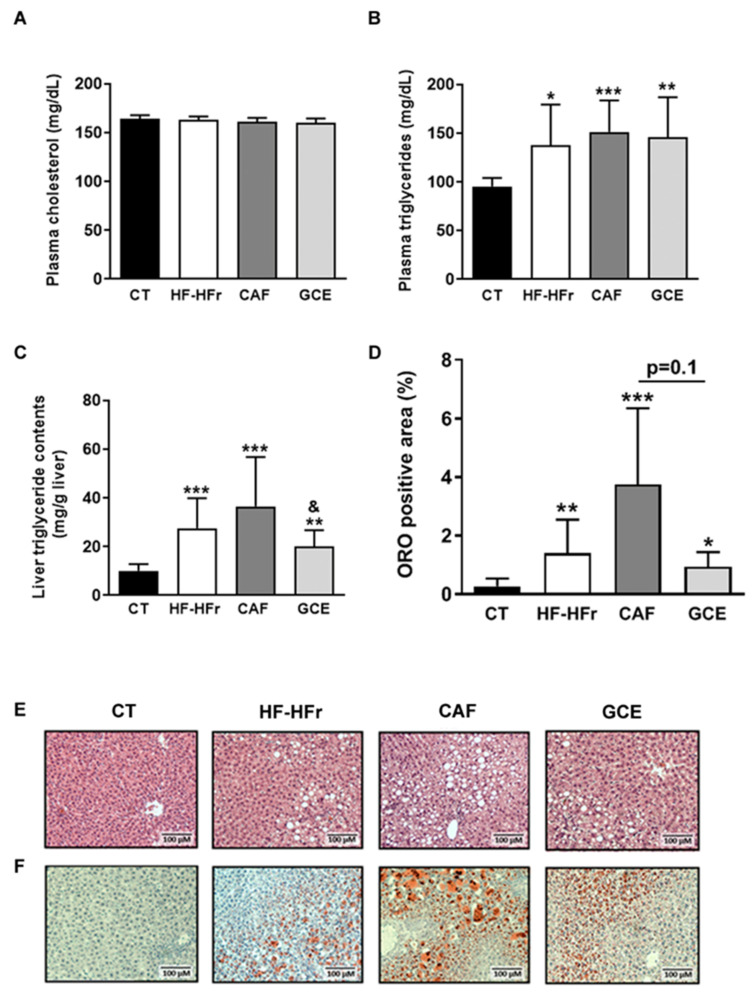
Blood cholesterol (**A**), blood triglyceride (**B**), and liver triglyceride (**C**) levels. Results are the mean ± SD of values from 10–12 animals/group. (**D**) Bar plot representing the mean ± SD percentage of area of positive Oil Red O staining calculated as positive stained area per total area section in CT, HF-HFr, CAF, and GCE experimental groups (*n* = 10–12/group). Representative hematoxylin and eosin (**E**) and Oil Red O (**F**) stained liver sections from the four experimental groups. * *p* < 0.05; ** *p* < 0.01; *** *p* < 0.0001 vs. control. ^&^ *p* < 0.05 vs. CAF group.

Blood cholesterol was unaffected by the diet or treatments, whereas blood TG levels were similarly increased in the HF-HFr, CAF, and GCE groups, compared to the control rats (Figure 2A,B).

The hepatic TG concentration was also increased in the HF-HFr group versus the CT group, and neither CAF or GCE attenuated this increase. Interestingly, hepatic TG levels were significantly lower in the GCE group than in the CAF group (Figure 2C). The same trend was observed in the liver sections stained with H&E and ORO, although the difference between the GCE and CAF groups was only marginally significant (*p* = 0.1) (Figure 2D–F).

**Figure 3:**
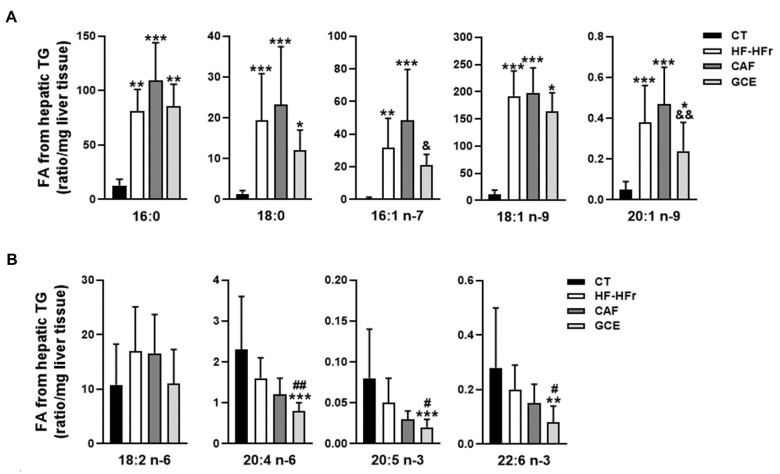
Fatty acid composition of hepatic triglycerides. (**A**) Saturated and monounsaturated fatty acids and (**B**) polyunsaturated fatty acids in the hepatic triglyceride fraction from CT, HF-HFr, CAF, and GCE experimental groups. Results are the mean ± SD of values from 9–10 animals/group. * *p* < 0.05; ** *p* < 0.01; *** *p* < 0.001 vs. control. ^#^ *p* < 0.05; ^##^ *p* < 0.01 vs. HF-HFr group. ^&^ *p* < 0.05; ^&&^ *p* < 0.01 vs. CAF group.

We also aimed to determine the fatty acid profile of the hepatic TGs. As shown in Figure 3A, the amount of SFAs [palmitic acid (16:0) and stearic acid (18:0)] and MUFAs [palmitoleic acid (16:1 n-7) and oleic acid (18:1 n-9)] in the hepatic TG fraction was strikingly increased by the HF-HFr diet. The addition of CAF or GCE did not significantly affect the levels of these SFAs compared to the HF-HFr group. Interestingly, the levels of both MUFAs were lower in the GCE group than in the CAF group, although the difference was significant only for palmitoleic acid. We also analyzed the levels of a less abundant MUFA in the TG fraction, 20:1 n-9, which showed also a significant increase in response to the HF-HFr diet and a decrease in the GCE group, compared to the CAF group (Figure 3A). Regarding PUFAs 20:4 n-6, 20:5 n-3, and 22:6 n-3, all showed lower levels in the GCE group than in the HF-HFr and the CT groups. The amount of linoleic acid (18:2 n-6) was not significantly altered by the diet or treatments (Figure 3B).

### 3.3. Liver Lipidomic Signatures Induced by the HF-HFr Diet and Effects of CAF and GCE

**Figure 4:**
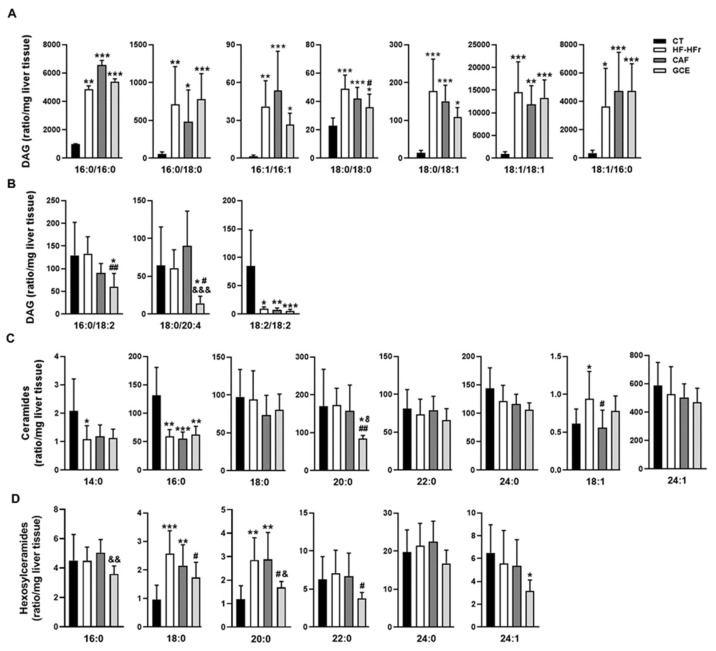
Lipidomic analysis in rat liver homogenates. Levels of diacylglycerols (DAG) (**A**,**B**), ceramides (Cer) (**C**), and hexosylceramides (HexCer) (**D**) in CT, HF-HFr, CAF, and GCE experimental groups. Results are the mean ± SD of values from 9–10 animals/group. * *p* < 0.05; ** *p* < 0.01; *** *p* < 0.001 vs. control. ^#^ *p* < 0.05; ^##^ *p* < 0.01 vs. HF-HFr group. ^&^ *p* < 0.05; ^&&^ *p* < 0.01; ^&&&^ *p* < 0.001 vs. CAF group.

Analysis of hepatic DAGs showed a striking effect of the HF-HFr diet, which increased the amount of SFA-, and MUFA-containing DAGs (Figure 4A). The addition of GCE to the HF-HFr diet significantly attenuated the increase in DAG 18:0/18:0 whereas CAF supplementation had no effect on this species. By contrast, the HF-HFr diet did not significantly increase the levels of PUFA-containing DAG (Figure 4B) and caused a large reduction in DAG 18:2/18:2. GCE treatment reduced the amount of DAG 16:0/18:2 and DAG 18:0/20:4.

We also analyzed the effect of diet and treatments on the amount of hepatic Cer and HexCer (Figure 4C,D). The HF-HFr diet significantly reduced the levels of Cer 14:0 and Cer 16:0. By contrast, the amount of Cer 18:1 was increased by the HF-HFr diet, with CAF significantly attenuating this increase. Similarly, HexCer 18:0 and HexCer 20:0 levels were increased in the HF-HFr group, with GCE attenuating the increases. Moreover, GCE exerted specific effects on several species that were not modified by the HF-HFr diet, such as the reduction of Cer 20:0 and HexCer 16:0, 22:0, and 24:1 levels.

As shown in Table 2, the ratio of HexCer 16:0, 18:0, 20:0, and 24:0 to the corresponding Cer was very low in the CT group and was increased by the HF-HFr diet. Again, we observed a differential effect of GCE, as this group showed lower HexCer/Cer ratios for 16:0 and 18:0 than the HF-HFr group, whereas CAF did not cause this effect.

### 3.4. Effects of the Diet and Treatments on the Fatty Acid Biosynthetic Pathway

**Figure 5:**
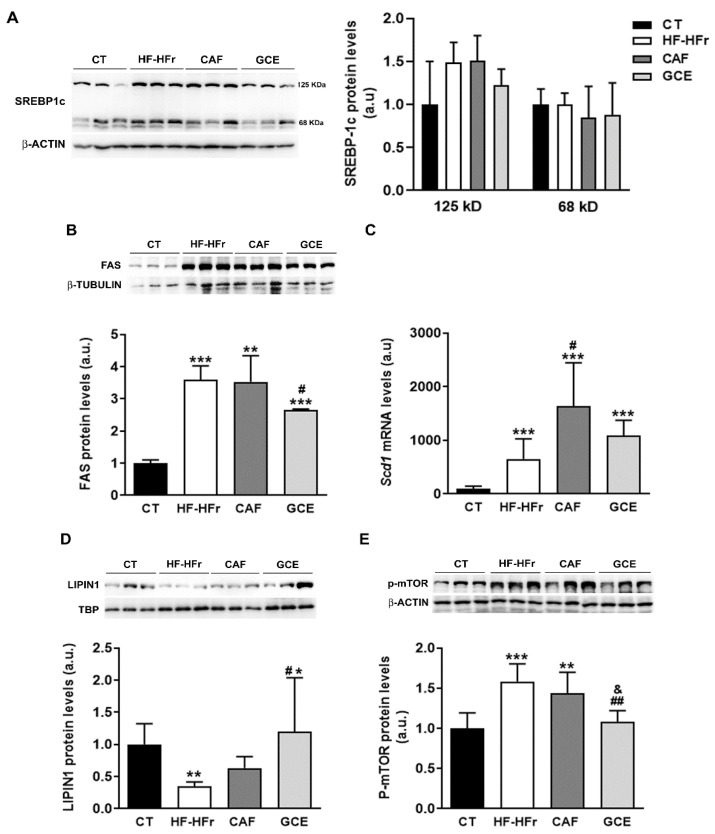
Western Blot of precursor (125 kD) and mature (68 kD) SREBP-1c (**A**) FAS: fatty acid synthase (**B**) lipin-1 (**D**) and phospho-mTOR proteins (**E**) in liver samples. Bar plots represent the mean ± SD band intensity of the proteins obtained from three samples per group, each one pooled from two animals. Bands are shown in the upper part of the figures. (**C**) Bar plot representing the mean ± SD mRNA levels corresponding to liver *Scd1* from CT (*n* = 5), HF-HFr (*n* = 6), CAF (*n* = 6), and GCE (*n* = 6) experimental groups. * *p* < 0.05; ** *p* < 0.01; *** *p* < 0.001 vs. CT. ^#^ *p* < 0.05; ^##^ *p* < 0.01 vs. HF-HFr. ^&^ *p* < 0.05 vs. CAF.

We determined the hepatic expression of sterol regulatory element-binding protein-1c (SREBP-1c), a transcription factor that controls the expression of enzymes involved in fatty acid synthesis. Both the precursor (125 kD) and the active form of SREBP-1c (68 kD) remained unaltered in the hepatic protein samples of all groups (Figure 5A). By contrast, the hepatic protein level of fatty acid synthase (FAS), a lipogenic enzyme controlled by this transcription factor, was increased significantly by the HF-HFr diet, with GCE partially preventing this increase (Figure 5B). The mRNA levels of another lipogenic enzyme controlled by SREBP-1c, stearoyl-CoA desaturase (*Scd1*), followed the same pattern of an increase in the HF-HFr group (Figure 5C). Interestingly, CAF increased *Scd1* expression even more than the HF-HFr diet, whereas GCE did not.

The observed effects of the diet on FAS and SCD1 expression suggested increased SREBP-1c transcriptional activity despite no changes in the amount of the active form of the protein. As shown in Figure 5D, the HF-HFr group showed a significant decrease in hepatic nuclear levels of lipin-1, which could modulate the transcriptional activity of SREBP-1c. Accordingly, the expression of phosphorylated mammalian target of rapamycin (P-mTOR), which phosphorylates lipin-1 and causes its nuclear exclusion, was increased in the livers of the rats from the HF-HFr group (Figure 5E). Interestingly, GCE relieved the reduction in lipin-1 levels caused by the diet, increasing the amount of this protein in nuclear extracts above CT levels (Figure 5D). Moreover, the GCE group returned P-mTOR levels to the control values, showing a significant reduction compared to the HF-HFr and CAF groups, which was in accordance with the increase in nuclear lipin-1 levels (Figure 5E).

### 3.5. CAF or GCE Does Not Affect Lipid Catabolic Pathways

**Figure 6:**
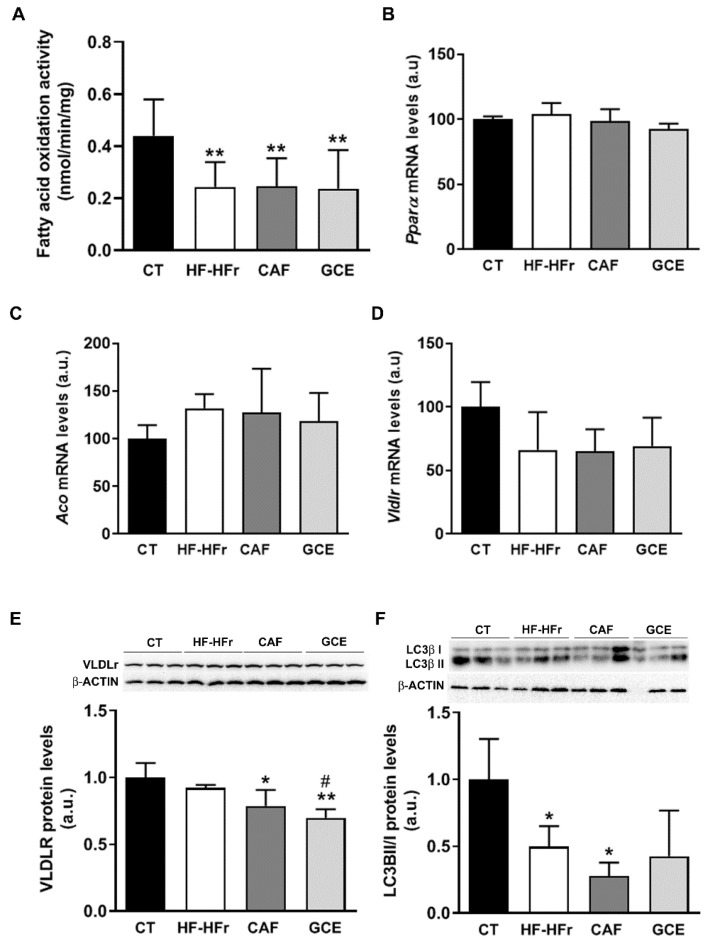

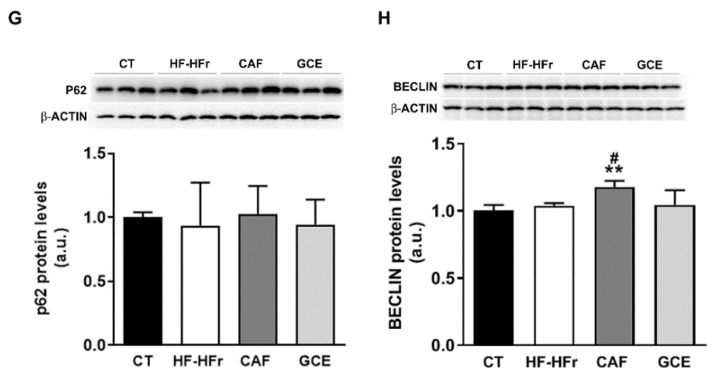
(**A**) β-oxidation activity in liver samples. Bars represent the mean ± SD of 10–12 samples per group. Bar plots representing the mean ± SD mRNA levels corresponding to liver *Pparα* (**B**), *Aco* (**C**), and *Vldlr* (**D**) genes from CT (*n* = 5), HF-HFr (*n* = 6), CAF (*n* = 6), and GCE (*n* = 6) experimental groups. Western Blot of VLDLR (**E**), LCII/I ratio (**F**), p62 (**G**), and beclin-1 (**H**) proteins, in liver samples obtained from CT, HF-HFr, CAF, and GCE experimental groups. Bar plots represent the mean ± SD band intensity of the proteins obtained from three samples per group, each one pooled from two animals. Bands are shown in the upper part of the figures. * *p* < 0.05; ** *p*< 0.01 vs. CT. ^#^ *p* < 0.05 vs. HF-HFr group.

To explore other mechanisms potentially involved in the observed effects on hepatic TGs, we determined the β-oxidation activity in liver samples. The results showed a significant decrease in response to the HF-HFr diet, with CAF or GCE addition having no effect on this decrease (Figure 6A). The mRNA levels of peroxisome proliferator-activated receptor α (*Pparα*), and the PPARα target genes acyl-CoA oxidase (*Aco*) and very-low density lipoprotein receptor (*Vldlr*) were not modified by the diet or treatments (Figure 6B–D). However, the protein levels of VLDLR despite not being increased by the HF-HFr diet were significantly lower in the CAF and GCE groups, and GCE even lowered the amount of this protein compared to the CAF group (Figure 6E).

The autophagy of lipid droplets was described as another form of lipid catabolism. As shown in Figure 6F, the ratio of the microtubule-associated protein 1A/1B-light chain 3 (LC3) B-II/I was significantly reduced in the HF-HFr group, with CAF or GCE treatment not reversing this decrease. However, neither diet nor treatments reduced the levels of the autophagy substrate p62 (Figure 6G), while beclin-1 levels showed a small but significant increase in the CAF group (Figure 6H).

### 3.6. Endoplasmic Reticulum Stress, Inflammation, and Oxidative Stress Markers

**Figure 7:**
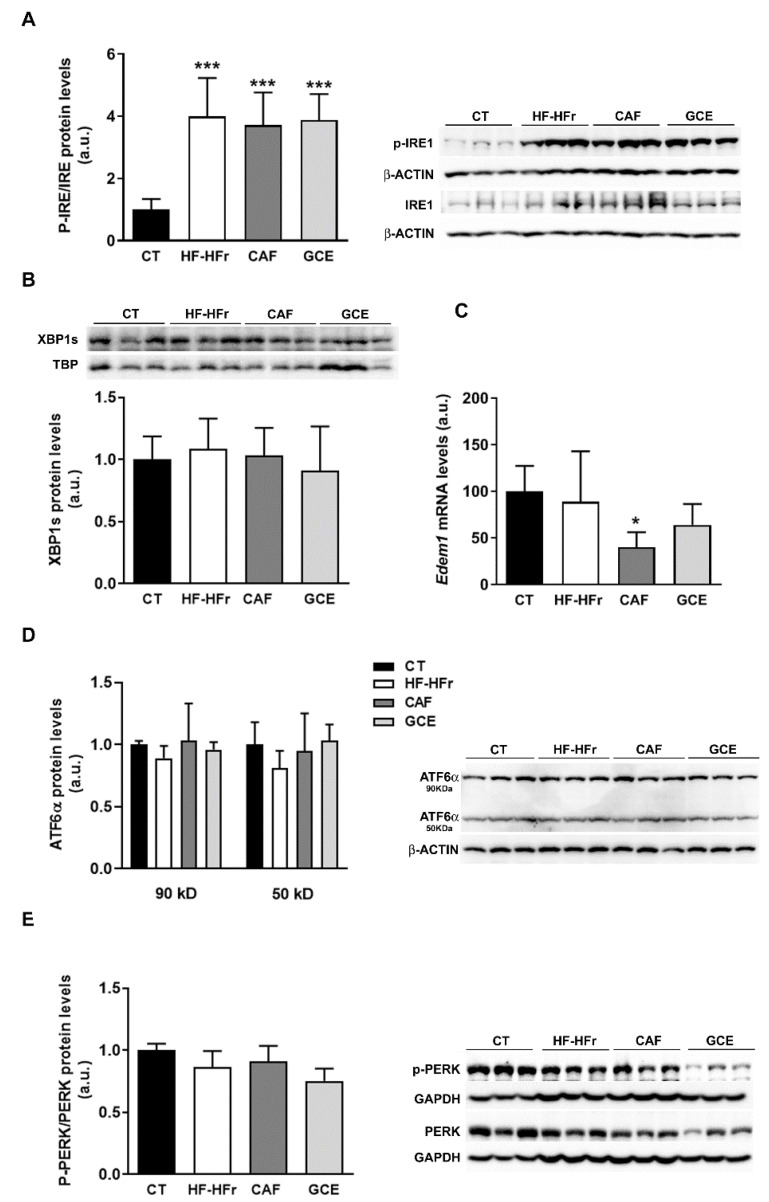
Western Blot of phosphorylated and total IRE1 (**A**), nuclear XBP1S (**B**), precursor (90 kD) and mature (50 kD) ATF6 (**D**), and phosphorylated and total PERK (**E**) in liver samples. Bar plots represent the mean ± SD band intensity of the proteins obtained from three samples per group, each one pooled from two animals. Bands are shown in the upper part of the figures. (**C**) mRNA levels of *Edem1* in the livers from CT (*n* = 5), HF-HFr (*n* = 6), CAF (*n* = 6), and GCE (*n* = 6) experimental groups. * *p* <0.05; *** *p* < 0.001 vs. CT.

We also explored other cell signaling pathways that could modulate hepatic lipid levels, such as endoplasmic reticulum (ER) stress. The HF-HFr diet significantly increased inositol-requiring enzyme-1α (IRE1α) phosphorylation, with neither CAF nor GCE reversing this increase (Figure 7A). However, levels of the active/spliced form of X-box-binding protein 1 (XBP-1s) protein in nuclear extracts were not significantly modified by any treatment, and mRNA levels of the XBP-1s target gene ER degradation-enhancing α-mannosidase-like 1 (*Edem1*) were not altered by HF-HFr diet and showed reduced expression in the CAF group (Figure 7B,C). Levels of the precursor (90 kD) and mature form (50 kD) of activating transcription factor 6 (ATF6) and phosphorylation of protein kinase RNA-like ER kinase (PERK) were not altered in any group (Figure 7D,E).

**Figure 8:**
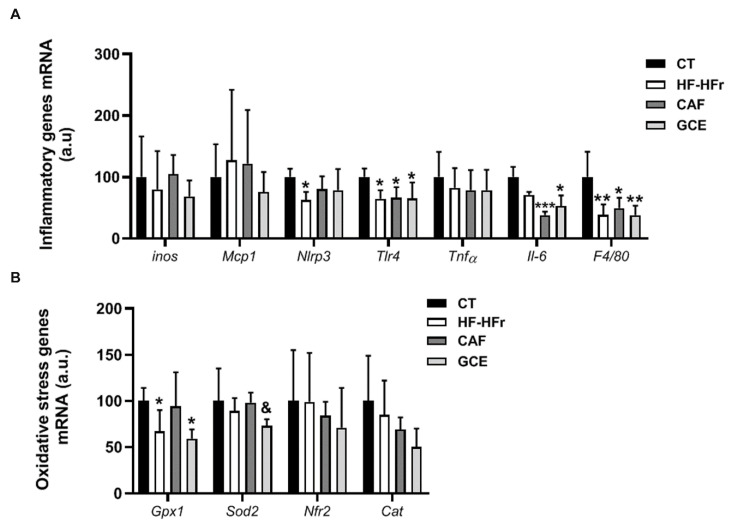
Bar plots showing the mean ± SD of specific mRNAs of pro-inflammatory molecules *iNos*, *Mcp1*, *Nlrp3*, *Tlr4*, *Tnfα*, *Il-6*, and *F4/80* (**A**) and oxidative stress genes *Gpx1*, *Sod2*, *Nrf2*, and *Cat* (**B**) in the livers from CT (*n* = 5), HF-HFr (*n* = 6), CAF (*n* = 6), and GCE (*n* = 6) experimental groups. * *p* < 0.05; ** *p* < 0.01; *** *p* < 0.001 vs. control. ^&^ *p* < 0.05 vs. CAF group.

Finally, we assessed the expression of several inflammation and oxidative stress markers. The experimental diet used did not induce an inflammatory response in the liver. In fact, the mRNA expression of several inflammation-related genes was reduced, with the treatments showing negligible effect (Figure 8A). In line with these results, the plasma levels of the inflammation marker ALT were not increased by the diet or treatments (Table 1). Similarly, the HF-HFr diet did not induce hepatic oxidative stress, and even reduced glutathione peroxidase 1 (*Gpx1*) expression. The GCE group showed lower mRNA levels of superoxide dismutase 2 (*Sod2*) compared to the CAF group (Figure 8B).

## 4. Discussion

Although nearly all rodent models on a high-fat diet rich in saturated fatty acids are characterized by obesity and insulin resistance [20], it is increasingly being recognized that a substantial proportion of individuals present NAFLD without obesity [2]. To obtain a model of NAFLD in its initial phase of simple hepatic steatosis, we fed female Sprague-Dawley rats a high-fat diet, which provides an exogenous source of fatty acids, and added liquid fructose (10% w/v) to their drinking water to promote de novo lipogenesis (DNL) [21]. To avoid the dietary intake of cholesterol, which is thought to activate Kupffer cells and stellate cells, and induce inflammation and fibrosis characteristic of NASH [22], we used cocoa butter instead of milk, as the source of saturated fatty acids in the high-fat diet.

Administration of the HF-HFr diet for three months caused hypertriglyceridemia and hepatic lipid deposition in the female Sprague-Dawley rats, but not inflammation, ER stress, or oxidative stress. Moreover, the rats fed the HF-HFr diet did not show an increase in body weight and adiposity, despite receiving around 1.8-times more calories than the control rats, which could not be ascribed to increased energy expenditure through spontaneous locomotor activity. Furthermore, although the rats on the HF-HFr diet responded to a glucose challenge with a higher insulin secretion, the increased insulin levels successfully controlled blood glucose levels and reduced the expression of hepatic gluconeogenic genes. This suggests that despite a decrease in the ISI, the hepatic glucose output was reduced, whereas in a typical situation of hepatic insulin resistance it would be increased [23].

The lipidomic analysis of liver samples from the rats offered some clues to explain these features of the HF-HFr diet. One of the most important bioactive lipids are ceramides, a class of sphingolipids involved in insulin resistance, inflammation, oxidative stress, and NAFLD development [24]. It was suggested that saturated fat derived from DNL or from the diet induces ceramide synthesis and insulin resistance [25]. However, we found that the hepatic levels of most ceramide species were not increased by the diet, which could be attributed to the absence of inflammation, as liver ceramides were reported to be increased in NASH, but not in simple steatosis in humans [26]. Remarkably, mice deficient in ceramide synthase 5 (CerS5), which exhibit lower hepatic levels of Cer 16:0, were protected from developing obesity and insulin resistance when fed a high-fat diet [27]. Therefore, the 50% reduction in hepatic Cer 16:0 levels in the HF-HFr group might help explain the absence of liver insulin resistance and obesity in rats fed this diet.

Having obtained a model of lean NAFLD with simple hepatic steatosis, we aimed to determine whether a moderate dose of caffeine, or a green coffee extract providing the same dose of caffeine, was effective in reducing the liver lipid burden. One of the major sources of caffeine in the human diet is coffee, which was reported to have beneficial effects on liver health [9]. However, a positive effect of coffee on NAFLD was not clearly established in human studies. Thus, a lower prevalence of NAFLD was associated with higher coffee intake in the NHANES study [28] and in some meta-analyses [10], but this association was not confirmed in other studies [29,30]. However, studies in animal models of diet-induced steatosis showed that several components of coffee, including caffeine, might be effective in reducing liver fat deposition [31]. The different outcomes from the human and animal studies might be due to the high doses of caffeine administered to laboratory animals, which in some studies were equivalent to 6 cups of coffee a day, much higher than the usual consumption in humans [32]. To provide a more realistic scenario, we treated rats with a low dose of caffeine (5 mg/kg/day), which after conversion based on body surface area was equivalent to 66 mg of caffeine for a 70-kg human being [15]. This amount roughly corresponded to 1 cup (20–25 mL) of espresso coffee per day, which was reported to contain 2.4 to 4.5 mg/mL of caffeine [33].

We observed that neither CAF nor GCE alleviated the hypertriglyceridemia and hepatic steatosis caused by the HF-HFr diet. However, rats treated with GCE exhibited lower levels of hepatic TG than those treated with CAF. When we analyzed the fatty acid composition of these TGs, we found that the amounts of palmitoleic acid and 20:1 *n*-9 were increased by the HF-HFr diet but were lower in the GCE group than in the CAF group. Palmitoleic acid is generated from palmitic acid through SCD1, which, together with FAS, are lipogenic enzymes regulated by SREBP-1c. The HF-HFr diet, despite not affecting the SREBP-1c levels, increased mTOR phosphorylation, which is known to phosphorylate and exclude lipin-1 from the nucleus [34]. This might lead to increased SREBP-1 transcriptional activity and, consequently, to FAS and SCD1 induction. Interestingly, the livers of the HF-HFr rats showed reduced nuclear levels of lipin-1 together with increased FAS and *Scd1* expression. These changes were not reversed neither by CAF nor by GCE. In fact, the expression of *Scd1* was higher in the CAF group than in the HF-HFr group, suggesting that CAF could further increase hepatic lipogenesis and worsen hepatic lipid deposition. However, neither the amount of hepatic TG nor lipin-1 or p-mTOR protein levels were different between the CAF and HF-HFr groups. In contrast, the GCE-treated rats showed lower mTOR phosphorylation and higher nuclear levels of lipin-1 than those of the rats from the HF-HFr group, suggesting lower SREBP-1 transcriptional activity. This might explain why FAS and SCD1 expression were induced to a lesser extent by GCE than CAF, and was in accordance with the lower levels of palmitoleic acid and total TGs observed in the livers of the GCE-treated rats.

The different effects of GCE compared to CAF were also observed with several DAG species, namely 18:0/18:0, 16:0/18:2, and 18:0/20:4, whose levels were reduced by GCE treatment compared to the HF-HFr group, but not by CAF treatment. Although there is a paucity of information about the effects of specific DAG species, it is generally assumed that DAGs play a role not only in insulin resistance but also in hepatic steatosis [35]. Therefore, the reduction of at least some of the DAGs accumulated in the liver might be regarded as a positive effect of other compounds contained in the GCE, given that caffeine alone did not cause such a reduction.

The hepatic levels of HexCer, which are formed from Cer by the enzyme glucosylceramide synthase (GCS), were reduced in the GCE group, as well as the 16_0 and 18_0 HexCer/Cer ratio. The HexCer/Cer ratio was considered to be an indicator of GCS activity. Interestingly, treatment of ob/ob mice with an inhibitor of GCS was reported to reduce TG accumulation in the liver [36]. Thus, the lower HexCer/Cer ratios observed in the GCE group might also be associated with reduced GCS activity and lower levels of liver triglycerides in this group, suggesting beneficial effects of GCE on hepatic steatosis.

To gain more insight into the mechanisms involved in the regulation of hepatic TG accumulation, we also examined several pathways linked to fatty acid catabolism. The reduced hepatic activity of β-oxidation could contribute to increased liver TG accumulation in the HF-HFr group. However, none of the treatments reversed this decrease, suggesting that reduced catabolism of fatty acids also occurred in the CAF and GCE groups. By contrast, the hepatic protein levels of VLDLR, which were reported to increase in animal models and humans with hepatic steatosis [37], were significantly reduced by GCE, compared to the CAF group, although they were not significantly modified by the HF-HFr diet. Reduced VLDLR levels could contribute, at least partly, to the lower hepatic TG accumulation observed in the GCE group.

Due to its role in lipid droplet degradation, autophagy is another mechanism that can lead to liver fat removal [38]. Our group previously showed that liquid fructose supplementation in female rats inhibit liver autophagy, as shown by the lower LC3II/I ratio, which leads to increased liver TG levels [39]. In the current study, we also observed a reduced LC3II/I ratio and TG accumulation in the livers of rats receiving the HF-HFr diet, suggesting inhibition of hepatic autophagy. CAF-treated rats showed the lowest LC3II/I ratio and the highest TG levels in the liver, which despite a slight increase in the beclin-1 protein levels indicated that CAF did not activate autophagy in our model. Other studies suggest that CAF induced autophagy in the liver [11], but they used higher doses of CAF (30 mg/kg/day compared to 5 mg/kg/day in our study).

In conclusion, a moderate dose of caffeine, equivalent to 1 cup of coffee a day in humans, did not alleviate liver lipid deposition in a model of diet-induced hepatic steatosis, without obesity and inflammation. One limitation of our study was that we did not treat rats fed a control diet, so we cannot rule out that caffeine could have exerted some effects in rats not exposed to HF-HFr. However, our goal was to investigate whether caffeine could reverse the hepatic steatosis induced by the HF-HFr diet. The lack of effect of caffeine in our study could be attributed to the duration of treatment, to the fact that treatment was initiated two months after the introduction of the HF-HFr diet or to the low dose used. However, when the same dose of caffeine was administered through a coffee extract, despite not normalizing the hepatic TG levels, these were lower than when the caffeine was administered alone. The coffee extract was rich in other compounds such as polyphenols, which might be responsible for the different effects observed. Vitaglione et al. showed that decaffeinated coffee reduced lipid droplet accumulation in hepatocytes, in a model of NASH, suggesting that caffeine was not essential for the anti-steatotic effect of coffee [40]. However, few studies compared the effects of caffeine with other coffee compounds on hepatic steatosis. A study conducted in mice concluded that only treatment with chlorogenic acid significantly reduced hepatic TG levels, whereas administration of pure caffeine did not [41]. Similarly, female mice treated with catechines or with catechines combined with caffeine, reduced liver TG levels, whereas caffeine alone did not [42]. In mice fed an HFD, administration of chlorogenic acid or caffeine alone did not reduce hepatic TG, but a combination of both compounds was effective [43]. Although the molecular mechanisms involved are not clearly established, this study suggest a synergistic effect on several pathways controlling fatty acid metabolism, including SREBP1c and lipogenic enzymes, such as SCD1 and FAS. Along the same lines, our results suggest that GCE components, either independently or in combination with CAF, might lead to: (i) less lipogenesis due to lower mTOR phosphorylation and higher nuclear levels of lipin-1, affecting FAS and SCD1 expression; (ii) a reduced amount of several DAG species; (iii) a lower HexCer/Cer ratio, which is a marker of GCS activity; and (iv) reduced expression of hepatic VLDLR. Although these changes are subtle, their combination might contribute to the different effects of the extract when compared to caffeine alone.

## Figures and Tables

**Table 1:** Zoometric parameters, blood analytes, and open field test results.

	CT (*n* = 12)	HF-HFr (*n* = 10)	CAF (*n* = 12)	GCE (*n* = 12)
Final body weight (g)	270 ± 13	271 ± 13	280 ±10	270 ± 12
Liver weight/body weight	2.9 ± 0.2	3.5 ± 0.4 **	3.8 ± 0.4 ***	3.6 ± 0.3 ***
vWAT weight/body weight	2.5 ± 0.7	3.1 ± 0.8	3.0 ± 0.6	2.9 ± 0.7
AUC consumed diet (Kcal/90 days/rat)	3884 ± 122	2728 ± 511 ***	2821 ± 352 ***	2710 ± 438 ***
AUC ingested liquid (Kcal/90 days/rat)	0	4098 ± 1201 ***	4330 ± 565 ***	4093 ± 750 ***
Total calorie intake (kcal/animal/90 days)	3884 ± 122	6827 ± 744 ***	7151 ± 141 ***	6803 ± 401 ***
Blood insulin (ng/mL)	1.2 ± 1.1	2.3 ± 1.0	3.3 ± 1.0	2.9 ± 1.4
Blood glucose (mg/dL)	117.2 ± 19.1	111.9 ± 12.7	120.2 ± 11.9	121.4 ± 15.3
ISI	1.1 ± 0.4	0.5 ± 0.2 *	0.5 ± 0.1 ***	0.6 ± 0.3 **
ALT (U/L)	19.9 ± 5.2	23.4 ± 5.2	19.8 ± 6.0	22.1 ± 5.8
Distance travelled in the OFT (cm)	8537 ± 1523	8429 ± 2110	8429 ± 1454	8316 ± 1985

Values are expressed as mean ± SD (*n* = 10–12). ALT: alanine aminotransferase; AUC: area under the curve; CAF: caffeine; CT: control; GCE: green coffee extract; HF-HFr: high-fat-high-fructose; ISI: insulin sensitivity index, calculated as 2/[plasma insulin (nM) × blood glucose (µM) + 1]; OFT: open field test; vWAT: visceral white adipose tissue. * *p* < 0.05; ** *p* < 0.01; *** *p* < 0.001 vs. control.

**Table 2:** Ratio hexosylceramide/ceramide.

	CT	HF-HFr	CAF	GCE
16_0	0.038 ± 0.017	0.076 ± 0.009 ***	0.093 ± 0.022 ***	0.059 ± 0.014 *** #
18_0	0.010 ± 0.004	0.028 ± 0.004 ***	0.030 ± 0.007 ***	0.021 ± 0.003 *** ^#^ &&
20_0	0.007 ± 0.002	0.016 ± 0.004 ***	0.019 ± 0.004 ***	0.019 ± 0.003 ***
22_0	0.076 ± 0.026	0.104 ± 0.049	0.082 ± 0.025	0.060 ± 0.015
24_0	0.139 ± 0.033	0.178 ± 0.040 *	0.191 ± 0.025 **	0.157 ± 0.024

Values are expressed as mean ± SD (*n* = 9–10). * *p* < 0.05; ** *p* < 0.01; *** *p* < 0.001 vs. CT. ^#^ *p* < 0.05 vs. HF-HFr; ^&&^ *p* < 0.01 vs. CAF.

## Supplementary Materials

The following are available online at https://www.mdpi.com/2072-6643/12/11/3240/s1, Supplemental Methods: Measurement of fatty acid methyl esters in liver TGs, Lipidomic analysis in rat liver homogenates. Table S1: Composition of the diets used in the study. Table S2: Primers used for RT-PCR. Table S3: GC-MS parameters for detection of fatty acid methyl esters using single ion monitoring. Table S4: Retention times and transitions for detection of lipids using LC-MS/MS.
