# Supplementary material for: Effects of a Low Dose of Caffeine Alone or as Part of a Green Coffee Extract, in a Rat Dietary Model of Lean Non-Alcoholic Fatty Liver Disease without Inflammation
Source: Nutrients. 2020 Oct 23;12(11):3240. doi: 10.3390/nu12113240 (PMC7690747; doi:10.3390/nu12113240)
Supplement: Supplementary file 1 [file nutrients-12-03240-s001.pdf]

Supplemental material for online publication

1. Supplemental Methods

Measurement of fatty acid methyl esters in liver TGs

Fatty acid methyl esters (FAMEs) from liver TGs were determined as follows. A weighed amount of liver (around 75 mg) was placed in a borosilicate glass tube (previously washed with n-hexane) containing 1 mL of 0.9% sterile sodium chloride. Tissue was homogenized on ice in two 10-second series with an OMNI TH Tissue Homogenizer (Omni International, Kennesaw, GA). The homogenate was spiked with 100 µL of the internal standard (ISTD) trinonadecanoin (100 µg/mL solution (10 µg); Nu-Chek Prep, Elysian, MN) and the lipids were extracted with 2 mL of chloroform/methanol (2:1 v/v). After centrifugation (5 min at 3,500 rpm), the organic phase was transferred to a new borosilicate glass tube and evaporated to dryness under N2 at 30°C. TGs were isolated by solid-phase extraction as described in Burdge et al[1]. Fatty acids were hydrolyzed and methylated following an adaptation of the method described by Agren et al [2]. Briefly, 100 µL of n-toluene and 500 µL of boron trifluoride-methanol solution (14%) were added to the tube, which was capped and placed into a block heater (100°C) for 60 min. After cooling, 500 µL of distilled water and 500 µL of n-hexane were added. The tubes were shaken for 1 min and then centrifuged for 5 min at 3,500 rpm at room temperature to separate the layers. The hexane layer was placed in a test tube and evaporated to dryness under a nitrogen stream (30°C and 15 psi). The extracts were reconstituted with 100 µL of n-hexane and transferred to an automatic injector vial containing a glass insert of 300 µL.

FAMEs were analyzed by gas chromatography/electron ionization mass spectrometry (GC/MS-EI), using an Agilent 6890N GC equipped with an Agilent 7683 autosampler, and an Agilent 5973N mass spectrometry detector. FAMEs were separated with a J&W DB-FastFAME capillary column (30 m × 0.2 mm × 0.25 µm film thickness) (Agilent). The injector temperature was set at 250°C, and 1 µL injections were performed (split ratio 25:1). GC was run using an optimized temperature program, as follows: the program started at 50°C, held for 0.5 min, increased to 194°C at a rate of 25°C/min, held for 1 min, increased to 245°C at a rate of 5°C/min, and held for 3 min. Helium was used as a carrier gas (14 psi, constant pressure mode). FAMEs were detected using the selected ion monitoring (SIM) mode. Based on the work of Thurnhofer and Vetter[3], several m/z ions common to saturated (SFA), monounsaturated (MUFA), and polyunsaturated (PUFA) FAMEs were monitored (see detailed information in Supporting information Table S3). Nine mixtures of FAME external calibration standards were prepared by diluting FAME mix certified reference material (Supelco 37 Component FAME Mix, Merck) in hexane. These standards were kept at -20°C until analysis. Thirty µL of each mixture were added to a tube, were spiked with 1000 µL of the ISTD C19:0-methyl ester (100 µg/mL solution (10 µg)), evaporated to dryness under a nitrogen stream (30°C and 15 psi), reconstituted with 100 µL of hexane, and transferred to an automatic injector vial containing a glass insert of 300 µL. The equivalents of C19:0 added to the samples as TG ISTD were the same as the amount of C19:0-methyl ester added to the external calibrators. The concentration of FAMEs in the samples were calculated by linear regression of the peak area ratio relative to that of the internal standard. The normalized concentrations were calculated by dividing the concentrations by the weight of the liver tissue.

Lipidomic analysis in rat liver homogenates

Levels of diacylglycerols [DAG], ceramides [Cer] and hexosylceramides [HexCer] in rat livers were determined as follows. In a borosilicate glass tube (previously washed with n-hexane),

the homogenate of a weighed amount of liver (around 75 mg), obtained as previously described, was spiked with 50 µL of an internal standard solution containing a mixture of 10 deuterated compounds supplied by Avanti Polar Lipids (see Supporting information Table S4 for further details). Lipids were subjected to a liquid-liquid extraction using 2 mL of chloroform/methanol (2:1 v/v). After centrifugation (5 min at 3500 rpm), 100 µL of the organic phase were transferred to a new tube (which had been previously washed with n-hexane) and evaporated in a water bath under a nitrogen stream (30°C and 15 psi). Extracts were reconstituted in 100 µL of the mobile phase consisting of methanol, transferred to HPLC vials and 10 µL were injected into the liquid chromatography-tandem mass spectrometry (LC-MS/MS) system.

Chromatographic separation of the lipid species was performed using an Acquity UPLC instrument (Waters Associates, Milford, Massachusetts, USA) operated using the MassLynx 4.1 software. The LC system was equipped with an Acquity UPLC® (BEH C18, 1.7 µm, 2.1 x 100 mm) column (Waters Associates). The flow rate was 0.3 mL/min and the temperature of the column was set at 55°C. An isocratic method was used, with a solution of 1 mM ammonium formate (NH4HCOO) and 0.01% HCOOH in methanol as the mobile phase solvent. The total run-time was 5 minutes. The detection of the ammonium adducts ([M + NH4]+ in the case of diacylglycerols) and protonated adducts ([M + H]+ for the other lipid species) was performed with a triple quadrupole (Xevo TQS-Micro MS, Waters) mass spectrometer equipped with an orthogonal Z-spray-electrospray ionization source (ESI) operated in the positive ion mode. The monitoring and quantification of the lipids was performed in the MRM mode using two different 5-minutes acquisition methods as detailed in Supporting information Table S4

Given that there are no commercially available standards for all the lipid species that were assessed in this study, the quantification of the compounds is expressed as a relative ratio and was calculated through dividing the peak area of the analyte by the peak area of the corresponding deuterated internal standard as specified in Supporting information Table S4. The normalized relative ratios were calculated by dividing the relative ratio by the weight of the liver tissue.

References

[1] G. C. Burdge, P. Wright, a E. Jones, S. a Wootton, *Br. J. Nutr.* **2000**, *84*, 781–787.

[2] J. J. Agren, A. Julkunen, I. Penttila, *J. Lipid Res.* **1992**, *33*, 1871–1876.

[3] S. Thurnhofer, W. Vetter, *J. Agric. Food Chem.* **2005**, *53*, 8896–8903.

2. Supplemental Tables

Table S1. Composition of the diets used in the study

High-fat diet. Teklad custom diet TD180456

	% by weight	% kcal from	Specific components	g/kg
Proteins	17.3	15.5	Casein	195.0
			L-Cystine	3.0
Carbohydrates	41.7	37.5	Corn Starch	336.9
			Maltodextrin	120.0
			Cellulose	50.0
Fat	23.2	46.9	Cocoa Butter	210.0
			Soybean Oil	20.0

Control diet. Teklad Global 14% Protein Rodent Maintenance Diet

	% by weight	% kcal from	Specific components	g/kg
Proteins	14.3	20		
Carbohydrates	48	67		
Fat	4	13		

Table S2. Primers used for RT-PCR

Gene	***GenBank™* nº**	***Primer sequences***	***PCR product***
Aco	NM_017340.2	Forward: 5'-GTGAGGCGCCAGTCTGAAA-3'<br>Reverse: 5'-ACTGCTGGGTTTGAAAATCCA-3'	70 bp
Cat	NM_012520.2	Forward: 5'-CGCCTGTGTGAGAACATTGC-3'<br>Reverse: 5'-TAGTCAGGGTGGACGTCAGT-3'	95 bp
Edem1	NM_001305279	Forward: 5'-TTGCCAGACGAGCTGTGAAA-3'<br>Reverse: 5'-TTTCCAACCCAATGGCCTGT-3'	100 bp
F4.80	NM_001007557.1	Forward: 5'- CAGATGGGGGATGACCACAC-3'<br>Reverse: 5'- AACTCTCCGTGGTGTCAGTG-3'	116 bp
Gpx1	NM_030826.4	Forward: 5'-TCGGTTTCCCGTGCAATC-3'<br>Reverse: 5'-TGAGGGAATTCAGAATCTCTTCATT-3'	69 bp
G6Pase	NM_013098.2	Forward: 5'-GGCTCACTTTCCCCATCAGG-3'<br>Reverse: 5'-ATCCAAGTGCGAAACCAAACAG-3'	146 bp
Il-6	NM_012589.2	Forward: 5'- ATATGTTCTCAGGGAGATCTTGGAA-3'<br>Reverse: 5'- TGCATCATCGCTGTTCATACAA-3'	79 bp
iNOS	NM_012611.3	Forward: 5'- CCCAGGAGGAGAGAGATCCG-3'<br>Reverse: 5'- AGCCATGACCTTCCGCATTA-3'	81 bp
Mcp1	NM_031530.1	Forward: 5'- CTGTCTCAGCCAGATGCAGTTAA-3'<br>Reverse: 5'- TGGGATCATCTTGCCAGTGA-3'	69 bp
Nlrp3	NM_001191642.1	Forward: 5'-GTGGAGATCCTAGGTTTCTCTG-3'<br>Reverse: 5'-CAGGATCTCATTCTCTTGGATC-3'	70 pb
Nrf2	XM_006234398.3	Forward: 5'-TGAAGACTGTATGCAGCTTTTGG-3'<br>Reverse: 5'-GGCAAGCGACTGAAATGTAGGT-3'	85 bp

Pepck	NM_198780.3	Forward: 5'-ACGGTGGGAACTCACTGCTTG-3'<br>Reverse: 5'-TGCCTTCGGGGTTAGTTATGC-3'	123 bp
Pparα	NM_013196.1	Forward: 5'- TGGAGTCCACGCATGTGAAG-3'<br>Reverse: 5'-CGCCAGCTTTAGCCGAATAG-3'	64 bp
Scd1	NM_139192.2	Forward: 5'- CAGAGCCAGGTGCCACTTTT-3'<br>Reverse: 5'-TGCTAGAGGGTGTACCAAGCTTT-3'	104 bp
Sod2	NM_017051.2	Forward: 5'-CGTCACCGAGGAGAAGTACCA-3'<br>Reverse: 5'-AGGCTGAAGAGCAACCTGAGTT-3'	70 bp
Tlr4	NM_019178.1	Forward: 5'-TTGTTCCTTTCCTGCCTGAGA-3'<br>Reverse: 5'-TGATCCATGCATTGGTAGGTAATATT-3'	83 bp
Tnf	NM_012675.3	Forward: 5'- ATCCGAGATGTGGAACTGGC-3'<br>Reverse: 5'- CGATCACCCCGAAGTTCAGTA-3'	151 bp
Vldlr	NM_013155.2	Forward: 5'-CCAGGAACAGGACTGCAGAG-3'<br>Reverse: 5'-GCCACCGTTATTGACCAAGC-3'	82 bp
Tbp	NM_001004198.1	Forward: 5'-TGGGATTGTACCACAGCTCCA-3'<br>Reverse: 5'-CTCATGATGACTGCAGCAAACC-3'	132 bp

Table S3. GC-MS parameters for detection of FAMEs using single ion monitoring.

Fatty acid	Retention time (minutes)	Quantifier ion (m/z)	Qualifier ion (m/z)
C16:0	8.0	74.0	87.0
C16:1	8.2	55.0	74.0
C18:0	9.3	74.0	87.0
C18:1n9cis	9.5	55.0	74.0
C18:2n6cis	9.9	81.0	67.0
C20:1n9	11.1	55.0	74.0
C20:4n6	12.2	79.0	91.0
C20:5n3	12.8	79.0	91.0
C22:6n3	15.1	79.0	91.0

Table S4. Retention times and transitions for detection of lipids using LC-MS/MS.

Lipid	Retention time (min)	Precursor m/z	Product m/z	Collision Energy (eV)	Internal standard	Acquisition method
DAG 16:0	1.9	586.2	313.0	20	DAG 16:0-D5	1
DAG 16:1	1.4	582.3	311.1	20	DAG 16:1-D5	1
DAG 16:0 18:2	1.7	610.2	313.2	20	DAG 16:0-D5	1
DAG 18:1 16:0	2.0	612.1	339.1	20	DAG 16:0-D5	1
DAG 16:0 18:0	2.4	614.2	313.1	20	DAG 16:0-D5	1
DAG 18:2	1.5	634.3	337.2	20	DAG 18:2-D5	1
DAG 18:0 18:2	2.1	638.1	341.0	20	DAG 16:0-D5	1
DAG 18:1	2.0	638.2	339.1	20	DAG 18:1-D5	1
DAG 18:0 18:1	2.4	640.2	341.2	20	DAG 18:0-D5	1
DAG 18:0	2.9	642.1	341.0	20	DAG 18:0-D5	1
DAG 18:0 20:4	2.0	662.2	341.3	20	DAG 20:4-D5	1
MAG 18:1	0.8	357.0	265.0	10	Cer 24:1-D7	1
MAG 18:2	0.7	355.0	263.0	10	DAG 18:2-D5	1
MAG 20:4	0.7	379.0	287.0	10	DAG 20:4-D5	1
LPC 16:0	0.7	496.3	184.0	20	Cer 16:0-D7	1
LPC 18:0	0.8	523.4	104.0	20	Cer 18:0-D7	1
SM 18:1	1.3	731.5	86.0	50	Cer 24:1-D7	1
Sphingosine	0.7	300.3	282.3	15	Cer 16:0-D7	2
Cer 18:1 14:0	1.2	510.3	264.3	30	Cer 16:0-D7	Both
Cer 18:1 16:0	1.4	538.2	264.3	30	Cer 16:0-D7	Both
Cer 18:1 18:0	1.6	566.2	264.3	30	Cer 18:0-D7	Both
Cer 18:1 20:0	1.9	594.2	264.3	30	Cer 18:0-D7	Both
Cer 18:1 22:0	2.3	622.6	264.3	30	Cer 24:0-D7	Both
Cer 18:1 24:0	2.8	650.5	264.3	30	Cer 24:0-D7	Both
Cer 18:1 18:1	1.4	654.2	264.3	30	Cer 24:1-D7	Both
Cer 18:1 24:1	2.3	648.3	264.3	30	Cer 24:1-D7	Both
HexCer d18:1 16:0	1.2	700.6	264.3	30	Cer 16:0-D7	2
HexCer d18:1 18:0	1.4	728.6	264.3	30	Cer 18:0-D7	2
HexCer d18:1 20:0	1.7	756.6	264.3	30	Cer 18:0-D7	2
HexCer d18:1 22:0	2.0	784.7	264.3	30	Cer 24:0-D7	2
HexCer d18:1 24:0	2.4	812.6	264.3	30	Cer 24:0-D7	2
HexCer d18:1 24:1	2.0	810.6	264.3	30	Cer 24:1-D7	2
DAG 16:0-D5	1.9	591.2	318.0	20	-	1
DAG 16:1-D5	1.4	587.3	316.2	20	-	1
DAG 18:0-D5	2.9	647.3	346.2	20	-	1
DAG 18:1-D5	2.0	643.4	344.3	20	-	1
DAG 18:2-D5	1.5	639.2	342.1	20	-	1
DAG 20:4-D5	1.4	687.0	366.0	20	-	1
Cer 18:1 16:0-D7	1.4	545.4	271.2	30	-	Both
Cer 18:1 18:0-D7	1.6	573.4	271.2	30	-	Both
Cer 18:1 24:0-D7	2.8	657.5	271.2	30	-	Both
Cer 18:1 24:1-D7	2.3	655.5	271.2	30	-	Both

Abbreviations: Cer: ceramide; DAG: diacylglycerol; HexCer: Hexosylceramide; MAG: monoacylglycerol; SM: sphingomyelin.
